# Mitochondrial LINC01133 regulates pyroptosis and calcium homeostasis in cisplatin-resistant lung adenocarcinoma

**DOI:** 10.20517/cdr.2026.39

**Published:** 2026-08-11

**Authors:** Feiyi Xu, Can Zhang, Sihan Wu, Yang Li, Yonggang Chen, Youwei Zhang

**Affiliations:** ^1^Department of Clinical Pharmacy, Xuzhou Central Hospital, Xuzhou 221009, Jiangsu, China.; ^2^Department of Oncology, Xuzhou Central Hospital, Xuzhou 221009, Jiangsu,China.; ^3^Department of Central Laboratory, Xuzhou Central Hospital, Xuzhou 221009, Jiangsu, China.

**Keywords:** LINC01133, cisplatin resistance, Caspase-1/GSDMD, pyroptosis, Ca^2+^ homeostasis, mitochondrial function

## Abstract

**Aim**: Cisplatin (DDP) represents the first-line chemotherapeutic agent for lung adenocarcinoma (LUAD), but acquired resistance severely limits its efficacy. This study investigated the role of the mitochondria-associated long non-coding RNA (mt-lncRNA) LINC01133 and its mechanisms in cisplatin resistance in LUAD.

**Methods:** LINC01133 expression and subcellular localization were detected through high-throughput mitochondrial RNA sequencing, quantitative reverse transcription-polymerase chain reaction (qRT-PCR), and fluorescence *in situ* hybridization (FISH). By modulating LINC01133 expression, we evaluated its effect on cisplatin resistance and malignant phenotypes. Further, western blotting, enzyme-linked immunosorbent assays (ELISAs), and calcium fluorescent probes were used to analyze how LINC01133 affects the Caspase-1/GSDMD signaling pathway, mitochondrial calcium homeostasis, and mitochondrial function. Finally, *in vitro* rescue experiments and a xenograft mouse model were conducted to validate the function of LINC01133 and its key targets.

**Results:** LINC01133 expression markedly increased in A549/DDP cells and was enriched in mitochondria. Clinical analysis revealed a difference in LINC01133 expression between LUAD and normal lung tissues, and higher LINC01133 expression was associated with poor patient survival. Functionally, LINC01133 overexpression enhanced cisplatin resistance and malignant phenotypes in A549 cells, whereas its knockdown reversed the resistance phenotype and inhibited malignant behaviors in A549/DDP cells. Mechanistically, cisplatin triggered the Caspase-1/GSDMD pathway to induce pyroptosis. LINC01133 inhibited activation of this pathway, alleviating GSDMD-NT-mediated mitochondrial calcium overload and thereby preserving mitochondrial structural integrity and function, ultimately enhancing cisplatin resistance.

**Conclusion:** LINC01133, a mitochondria-enriched oncogenic lncRNA, promotes cisplatin resistance in LUAD by inhibiting the Caspase-1/GSDMD pathway and maintaining mitochondrial calcium homeostasis and function.

## INTRODUCTION

Lung cancer remains a major cause of cancer-associated mortality worldwide, and lung adenocarcinoma (LUAD) is the most predominant non-small cell lung cancer (NSCLC) subtype^[1,2]^. Although immunotherapy, represented by PD-1/PD-L1 inhibitors, is extensively adopted clinically, cisplatin-based chemotherapy remains the mainstream first-line therapy and holds an irreplaceable position in the comprehensive management of advanced-stage patients^[3-6]^. Nonetheless, resistance to cisplatin severely curtails its clinical efficacy, which represents an important factor leading to treatment failure and poor patient prognosis^[7,8]^. Consequently, it is highly important to elucidate cisplatin resistance-related mechanisms and develop new solutions to improve LUAD patient prognosis.

The functional status of mitochondria, the core organelles that govern energy metabolism and cell death regulation, directly influences the sensitivity of tumor cells to chemotherapeutic agents. Tumor cells can evade chemotherapy-induced cell death via diverse mitochondria-related mechanisms, such as reprogramming oxidative phosphorylation, regulating reactive oxygen species (ROS) homeostasis, increasing the accumulation of mitochondrial DNA (mtDNA) mutations, altering membrane potential, as well as disrupting mitochondrial dynamics^[9-11]^. Consequently, adaptive changes in mitochondrial function are considered a core mechanism of acquired drug resistance. Cisplatin primarily exerts its antitumor effect by generating DNA crosslinks to initiate apoptosis^[12]^, and tumor cells can acquire a resistant phenotype by remodeling mitochondrial function to circumvent death signals. Research has confirmed that mitochondrial dysfunction - such as enhanced oxidative metabolism, reduced ROS levels, downregulated mtDNA expression, and disrupted mitochondrial dynamics - can significantly promote the development of cisplatin resistance in various malignancies^[13-17]^. These findings highlight that an in-depth understanding of the regulatory network governing mitochondrial functional homeostasis is crucial for revealing the mechanisms through which tumor cells acquire cisplatin resistance.

Long non-coding RNAs (lncRNAs) can specifically interact with nucleic acids and proteins, readily respond to cellular stress signals, play key roles in nuclear-mitochondrial communication, and finely regulate mitochondrial metabolism and cell fate^[18-20]^. Our research team has previously conducted systematic studies concerning the effects of mt-ncRNAs on cisplatin resistance in LUAD. High-throughput sequencing of mitochondrial RNA from parental A549 cells and their cisplatin-resistant counterpart A549/DDP revealed several differentially expressed mitochondria-associated lncRNAs, including LINC01133. However, unlike the mechanism through which H19 regulates mitophagy^[21]^, the biological function of LINC01133 and its specific role in cisplatin resistance remain unclear.

## METHODS

### Cell lines and cell culture

Parental A549 and PC9 human LUAD cells were purchased from Wuhan Servicebio Technology Co., Ltd. (Wuhan, China), whereas cisplatin-resistant A549/DDP cells were purchased from Wuhan Pricella Biotechnology Co., Ltd. (Wuhan, China). All cell lines were cryopreserved and stored in the Central Laboratory of Xuzhou Central Hospital. According to the suppliers’ product information, these cell lines were authenticated by short tandem repeat profiling and were negative for mycoplasma contamination. A549 and A549/DDP cells were cultured in high-glucose DMEM supplemented with 10% premium fetal bovine serum and 1% penicillin-streptomycin, whereas PC9 cells were cultured in RPMI-1640 medium containing the same supplements. All cells were maintained at 37 °C with 5% CO_2_. Cells between passages 5 and 15 after thawing were used for all experiments. According to the supplier, the cisplatin-resistant A549/DDP subline was established from parental A549 cells through stepwise selection with increasing concentrations of cisplatin. Following thawing, the cells were allowed to recover in cisplatin-free medium and were subsequently exposed to progressively increasing concentrations of cisplatin (0.4, 0.8, 1.2, 1.6, and 2.0 μg/mL) to restore selective pressure and maintain the resistant phenotype. Once stable growth in the presence of 2.0 μg/mL cisplatin was achieved, the cells were routinely maintained in medium containing the same concentration of cisplatin. Before the experiments, A549/DDP cells were cultured in cisplatin-free medium for 48 h to minimize the potential acute effects of the maintenance drug.

### Cell Counting Kit-8

Cells (5 × 10^3^/well) were seeded into 96-well plates. Under light-protected conditions, Cell Counting Kit-8 (CCK-8) reagent (10 μL; Dojindo, CK04) was added to each well, and the cells were then incubated for 1 h at 37 °C. Optical density (OD) was measured with a multifunctional microplate reader at 450 nm. The half-maximal inhibitory concentration (IC_50_) was calculated from the OD values, and the resistance index (RI) was calculated as follows: RI = IC_50_ of drug-resistant cells/IC_50_ of parental cells.

### Mitochondrial isolation and extraction of mitochondrial RNA

A cell mitochondria isolation kit (Beyotime, C3601) was used for mitochondrial isolation. Briefly, 1 mL of mitochondrial isolation reagent was added to 2 × 10^7^ cells, followed by incubation on ice for 15 min. Subsequently, the cell suspension was transferred to a glass homogenizer and manually homogenized about 30 times. Purified mitochondria were collected through density gradient centrifugation. RNA-easy isolation reagent (Vazyme, R701) was used to extract RNA from the mitochondrial pellet through phase separation, precipitation, and washing steps. The extracted RNA was thoroughly mixed with DEPC-treated water before storage at -80 °C.

### Quantitative reverse transcription-polymerase chain reaction

RNA was reverse transcribed into cDNA using SweScript All-in-One RT SuperMix for qPCR (ServiceBio, G3337). Using cDNA as the template, qPCR was performed with 2× Universal Blue SYBR Green qPCR Master Mix (ServiceBio, G3326). The reaction conditions were as follows: 95 °C for 30 s, 95 °C for 15 s, and 60 °C for 30 s, repeated for 40 cycles. The primer sequences were designed and synthesized by Wuhan Servicebio Technology Co., Ltd., and the specific sequences are listed in Table 1.

**Table 1:** Primer sequences used for qRT-PCR

**Genes**	**Forward (5′-3′)**	**Reverse (5′-3′)**
GAPDH	GGAAGCTTGTCATCAATGGAAATC	TGATGACCCTTTTGGCTCCC
LINC01133	AACCTTTGCTCCAACTTTCTCCT	CTCTTTACCTCCTCCCAACCATT

qRT-PCR: Quantitative reverse transcription-polymerase chain reaction.

### Fluorescence *in situ* hybridization

Probes targeting LINC01133 were synthesized by Wuhan Servicebio Technology Co., Ltd., and its target sequence is listed in Table 2.

**Table 2:** Probe sequences used for LINC01133 FISH

	**Target sequence (5′-3′)**
**Probe 1**	GGGAAGCTAAGGAGAAAGTTGGAGCA
**Probe 2**	AGACTTAAGGTAGAGCCAGGCTGT
**Probe 3**	CACTGATGTTGGGCTGGAACAAGGACT
**Probe 4**	TCTTTACCTCCTCCCAACCATTCCC

FISH: Fluorescence *in situ* hybridization.

After fixation with 4% paraformaldehyde and permeabilization with 0.1% Triton X-100, the cell slides were pre-hybridized for 1 h at 37 °C and hybridized with a 500 nM LINC01133 probe overnight at 37 °C in the dark. The slides were then sequentially washed, hybridized, and subjected to signal amplification with 500 nM branch probes (40 °C, 45 min) and 1:200 diluted 488-labeled signal probes (42 °C, 3 h). Then, 3% BSA was added to block the slides for 30 min, followed by overnight incubation with MitoTracker Orange CMTMRos (1.5 μM; Thermo Fisher, M7510) at 4 °C. Finally, DAPI was added for nuclear counterstaining. After the slides were mounted, images were acquired using a fluorescence microscope.

### Cell transfection and experimental groups

Lentiviral vectors for LINC01133 overexpression and RNA interference (RNAi) were prepared by Shanghai GeneChem Co., Ltd. The LINC01133-targeting siRNA sequences are listed in Table 3.

**Table 3:** siRNA sequences targeting LINC01133

	**Target sequence (5′-3′)**
**si-LINC01133-1**	GGAGCCATTAACAAAGCTTTC
**si-LINC01133-2**	GCCCAACATCAGTGCCTCTGA
**si-LINC01133-3**	GGCATAGGGATCCATTTATCC

Cells (1 × 10^5^/well) were seeded into 6-well plates and transfected the following day. After 48 h, the cells were selected using 2 μg/mL puromycin to establish stable LINC01133-overexpressing A549 cells (A549-OE-LINC01133) and stable LINC01133-knockdown A549/DDP cells (A549/DDP-sh-LINC01133). In addition, negative control groups infected with empty vector virus (A549-Vector, A549/DDP-NC) and uninfected blank control groups (A549-Control, A549/DDP-Control) were established. The same LINC01133-overexpressing lentiviral vector and corresponding empty vector were used to establish stable PC9-OE-LINC01133 and PC9-Vector cells, respectively, followed by puromycin selection.

### Transwell assay for migration and invasion

Complete medium (600 μL) containing the drug was added to the lower chamber of the 24-well plate, while the cell suspension (100 μL, 1 × 106 cells/mL) was added to the upper Transwell chamber. In the invasion assay, after dilution, Matrigel (Becton, 354230) was precoated onto the bottom of the upper chamber, followed by gelation at 37 °C for 3 h and hydration with serum-free medium for 30 min. After incubation, fixation, staining, and washing, the nonpenetrated cells in the upper chamber were gently removed using a cotton swab, and the samples were observed and recorded under a microscope.

### Flow cytometry analysis

After the cell samples were rinsed with phosphate buffered saline (PBS), an Annexin V-FITC/PI apoptosis detection kit (KeyGEN, K231205) was used for staining. The cell suspension was then filtered through a 200-mesh sieve before analysis using a flow cytometer.

### Transmission electron microscopy

Following fixation with 2.5% glutaraldehyde and 1% osmic acid, the cells were dehydrated with a gradient ethanol series, incubated with a mixture of 100% acetone and epoxy resin embedding medium (1:1 ratio) at ambient temperature for 2 h, transferred to pure embedding medium, and polymerized overnight at 70 °C. After ultrathin sections were prepared, they were double-stained with uranyl acetate and lead citrate. Finally, they were visualized using transmission electron microscopy (TEM).

### Extract cellular protein, tissue protein, and mitochondrial protein

Cells from 6-well plates and shredded tissue samples (homogenized with a glass homogenizer) were lysed with RIPA Lysis Buffer (Beyotime, P0013B) on ice. After centrifugation and collection of the supernatant, total cellular and tissue proteins were obtained. For mitochondrial protein extraction, the isolated mitochondrial pellet was lysed on ice for 20 min with 150 μL of mitochondrial lysis buffer (including 1 mM PMSF), followed by centrifugation and collection of the supernatant to obtain mitochondrial proteins.

### Western blotting

Protein samples were analyzed using a BCA protein assay kit (Beyotime, P0012). Protein aliquots (30 μg) were separated by 12% SDS-PAGE (Vazyme, E304) and transferred to PVDF membranes using wet transfer. After blocking with 5% defatted milk in TBST for 2 h at ambient temperature, the membranes were incubated with diluted primary antibodies overnight at 4 °C. The primary antibodies used were as follows: anti-Caspase 1 (Proteintech, 81482-1, 1:500), anti-Cleaved Caspase 1 (Affinity, AF4005, 1:500), anti-GSDMD (Proteintech, 80918-1, 1:1,000), anti-GSDMD-NT (Abcam, ab215203, 1:1,000), anti-TOMM20 (Servicebio, GB111481, 1:1,000), anti-COX IV (Servicebio, GB11250, 1:1,000), and anti-GAPDH (Servicebio, GB15004, 1:3,000). The following day, the membranes were incubated with an HRP-labeled goat anti-rabbit IgG secondary antibody (Servicebio, GB23303, 1:5,000) for 1 h. Protein bands were visualized using an enhanced chemiluminescence (ECL) kit (NCM, P10100) on a Bio-Rad ChemiDoc imaging system. The gray values of the protein bands were analyzed using ImageJ software. The relative expression level of the target protein was expressed as the ratio of the gray value of the target protein band to that of the internal reference protein band. For each biological replicate, protein samples were independently prepared from a separate cell culture experiment, and *n* = 3 represents three independent biological replicates.

### Enzyme-linked immunosorbent assays

Human IL-18 (MULTI SCIENCES, EK118/2) and IL-1β (Thermo Fisher, 88–7261) uncoated enzyme-linked immunosorbent assay (ELISA) kits were used to measure IL-18 and IL-1β levels in the cell supernatants. Briefly, cytokine standards were serially diluted to generate standard curves for IL-18 and IL-1β. Absorbance was measured at 450 nm using a microplate reader. The absolute concentrations of IL-18 and IL-1β in each sample were calculated based on the corresponding standard curves and expressed as ng/mL or pg/mL.

### Lactate dehydrogenase release assay

Using a lactate dehydrogenase (LDH) Cytotoxicity Assay Kit (Beyotime, C0016), the LDH concentration was determined according to the specified protocols. A microplate reader was used to measure the absorbance at 490 nm.

### Cellular and mitochondrial Ca^2+^ content assessment

Cells were seeded in black 96-well plates with clear bottoms. To assess mitochondrial and intracellular Ca^2+^ levels, cells were incubated with working solutions of Rhod-2 AM (Beyotime, S1062M) and Fluo-4 AM (Beyotime, S1060) in the dark. After probe loading and hydrolysis, fluorescence intensities were measured using a multifunctional microplate reader (mitochondrial Ca^2+^: Ex/Em = 549/578 nm; intracellular Ca^2+^: Ex/Em = 488/515 nm). For visualization, the cells were incubated with a combined staining working solution containing Rhod-2 AM (4 μM), MitoTracker Green (1 μM), and Hoechst 33342 (1 μM) at 37 °C in the dark for 20 min. Changes in mitochondrial Ca^2+^ were observed using a confocal microscope after the slides were mounted.

### Mitochondrial membrane potential assay (with JC-1)

A mitochondrial membrane potential assay kit with JC-1 (Solarbio, M8650) was used to measure changes in the mitochondrial membrane potential. The fluorescence of JC-1 monomers and aggregates was measured at Ex/Em = 490/530 and 525/590 nm, respectively, using a microplate reader.

### Mitochondrial ROS assessment

To assess mitochondrial ROS levels, 100 μL of MitoSOX™ Red mitochondrial superoxide indicator (Thermo Fisher, M36007) was added to the wells and incubated at 37 °C for 30 min in the dark. After the cells were rinsed twice with PBS, fluorescence intensities were measured using a multifunctional microplate reader (Ex/Em = 396/610 nm).

### Adenosine triphospate analysis

Mitochondrial adenosine triphospate (ATP) content was determined after mitochondrial lysis and centrifugation using an Enhanced ATP Assay Kit (Beyotime, S0027). ATP content was measured using a luminometer on a fluorescence microplate reader.

### mtDNA analysis

Total cellular DNA was extracted using a Universal Genomic DNA Purification Mini Spin Kit (Beyotime, D0063) in accordance with the specified protocols. PCR was performed to determine the relative mtDNA copy number, which was calculated as the ratio of the mitochondrial gene MT-CO1 to the nuclear gene B2M. The specific sequences are listed in Table 4.

**Table 4:** Primer sequences used for mtDNA copy number analysis by PCR

**Genes**	**Forward (5′-3′)**	**Reverse (5′-3′)**
MT-CO1	CTTCGTCTGATCCGTCCTCTAATC	TTGAGGTTGCGGTCTGTTAG
B2M	CCCACTGTCACATGCATTACT	GGTTGAGTTGGACCCGATAAA

mtDNA: Mitochondrial DNA; PCR: polymerase chain reaction.

### *In vivo* mouse xenograft assays

All animal experiments were approved by the Laboratory Animal Management and Use Committee of the Experimental Animal Center of Wuhan Servicebio Technology Co., Ltd. (Approval No. Saiwei’er Animal [Fu] No. 2025207). The 5-week-old female BALB/c nude mice used in the present study were maintained in a specific pathogen-free (SPF) environment. Following acclimatization, mice were randomly assigned to six groups using a random-number method (*n* = 6). Investigators responsible for drug administration were aware of the group allocation. To minimize assessment bias, tumor measurements, tumor weighing, and quantitative histological analyses were performed under coded, blinded conditions by investigators who were unaware of the group assignments. Group identities were disclosed only after completion of data analysis. Cells in the logarithmic growth phase were harvested and resuspended at a density of 1 × 10^7^ cells/mL. Each mouse was subcutaneously inoculated in the right dorsal flank with 0.1 mL of the cell suspension. One week after inoculation, following confirmation of tumor formation, the mice received intraperitoneal injections of cisplatin (DDP; 5 mg/kg), disulfiram (DSF; 75 mg/kg), or lipopolysaccharide (LPS; 50 μg/kg), as appropriate for the assigned group, once every 3 days for 4 consecutive weeks. During the treatment period, tumor length (a) and width (b) were measured every 3-4 days using digital calipers, and tumor volume was calculated according to the formula V = a × b^2^/2. Body weight was recorded concurrently using an electronic balance. Following the final treatment, the mice were euthanized by cervical dislocation, and the tumors were excised and weighed. A portion of each tumor was immediately snap-frozen in liquid nitrogen and stored at -80 °C for quantitative reverse transcription-polymerase chain reaction (qRT-PCR) analysis. The remaining tissues were fixed, embedded in paraffin, sectioned, and subjected to immunohistochemical (IHC) and terminal deoxynucleotidyl transferase dUTP nick-end labeling (TUNEL) staining.

### Statistical analysis

Experimental results were obtained from three or more independent replicates and are represented as the mean ± standard deviation. Normally distributed data with equal variance were compared between groups using Student’s *t* test and among groups using one-way analysis of variance (ANOVA) followed by the LSD post hoc test. Non-parametric data were analyzed using the Mann-Whitney *U* test. The log-rank test was applied to analyze survival, while multifactorial designs were examined using multifactorial ANOVA. All statistical analyses and visualizations were performed using GraphPad Prism 9.5 software, with *P* < 0.05 indicating statistical significance.

## RESULTS

### LINC01133 is upregulated and shows a mitochondria-enriched distribution in A549/DDP cells

**Figure 1:**
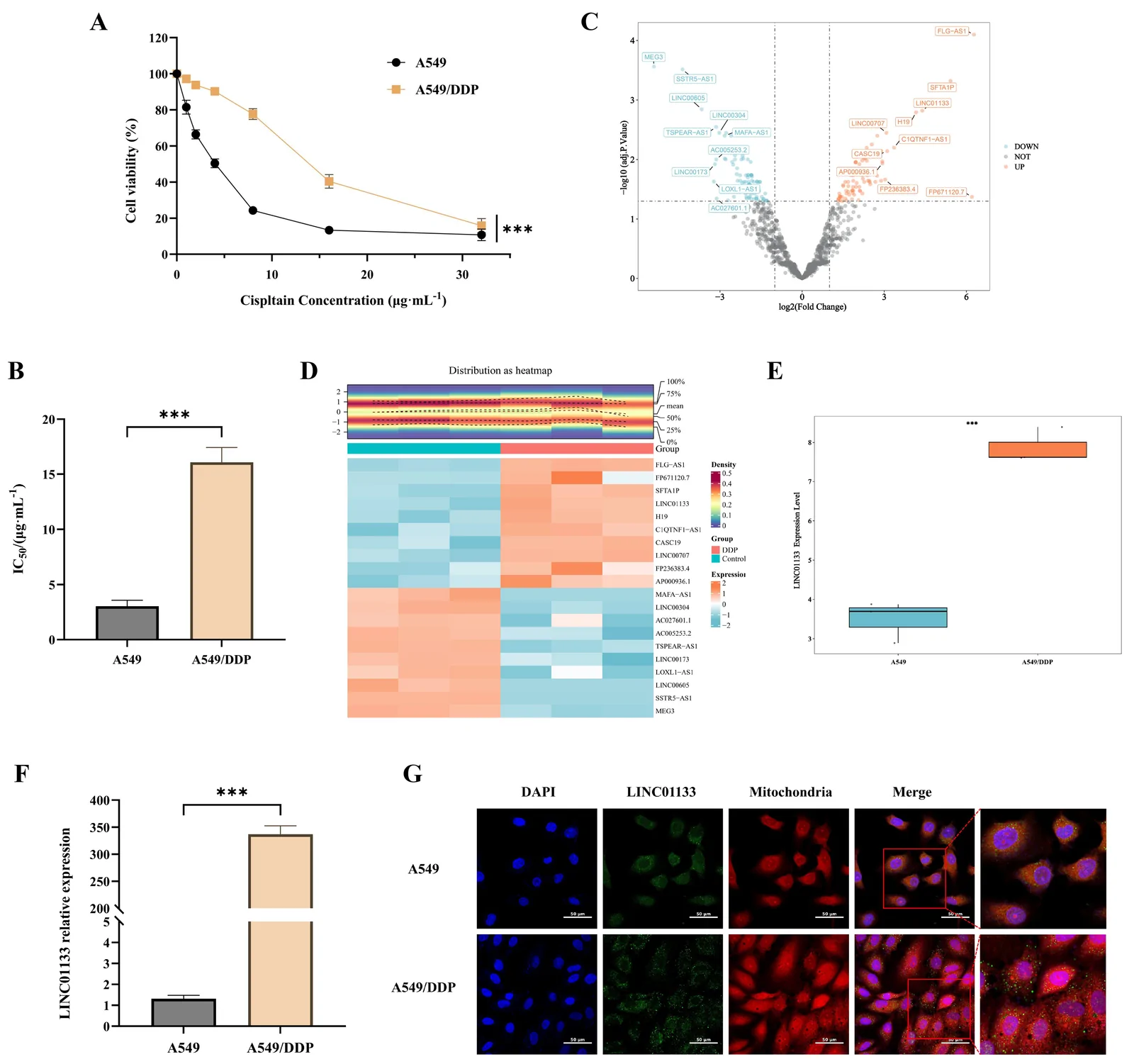
LINC01133 is upregulated and shows a mitochondria-enriched distribution in A549/DDP cells. (A) The viability of A549 and A549/DDP cells after treatment with different doses of cisplatin (0, 1, 2, 4, 8, 16, and 32 μg/mL) was measured by a CCK-8 assay; (B) Statistical analysis of the IC_50_ values for A549 and A549/DDP cells; (C) Volcano plot analysis of differentially expressed mt-lncRNAs in A549 *vs.* A549/DDP cells with |log_2_FC| ≥ 1 and adjusted *P* < 0.05 thresholds. Orange dots represent lncRNAs whose expression is significantly upregulated in A549/DDP cells, blue dots represent lncRNAs whose expression is significantly downregulated, and gray dots indicate genes whose expression is not significantly different; (D) Hierarchical clustering heatmap showing differentially expressed mt-lncRNAs between A549 and A549/DDP cells. Different colors indicate relative gene levels, with blue and red representing low and high expression, respectively; (E) Comparison of LINC01133 levels in the mitochondria of both cell lines based on transcriptome sequencing data; (F) Validation of relative LINC01133 expression in the mitochondria of both cell lines by qRT-PCR; (G) Subcellular localization of LINC01133 detected by FISH. Nuclei were stained with DAPI (blue), LINC01133 was visualized with a specific probe (green), and mitochondria were labeled with MitoTracker (red). Scale bar: 50 μm. Nonlinear regression analysis was conducted to fit the dose-response curves. Student’s *t*-test was adopted for comparing two-group means. ^***^P < 0.001. DDP: Cisplatin; CCK-8: Cell Counting Kit-8; IC_50_: half-maximal inhibitory concentration; mt-lncRNAs: mitochondria-associated long non-coding RNAs; qRT-PCR: quantitative reverse transcription-polymerase chain reaction; FISH: fluorescence *in situ* hybridization; DAPI: 4′,6-diamidino-2-phenylindole.

The results of the CCK-8 assay revealed that relative to A549 cells, A549/DDP cells had markedly increased viability when treated with the same concentrations of cisplatin, verifying that these cells had a stable drug-resistant phenotype [Figure 1A and B]. Based on our group’s established method for mitochondrial RNA isolation and sequencing, combined with the results of a qRT-PCR assay, the LINC01133 mRNA level markedly increased within the mitochondria of A549/DDP cells compared with that in A549 cells [Figure 1C-F]. Subcellular localization using fluorescence *in situ* hybridization (FISH) revealed that the fluorescence signal for LINC01133 was markedly enhanced in A549/DDP cells and was accompanied by a high degree of colocalization with mitochondrial markers [Figure 1G]. Taken together, these results indicate that LINC01133 is upregulated in cisplatin-resistant A549/DDP cells and exhibits a mitochondria-enriched distribution pattern, suggesting that it may participate in mitochondria-related mechanisms underlying cisplatin resistance in LUAD.

### LINC01133 expression differs between LUAD and normal lung tissues and is associated with poor patient survival

**Figure 2:**
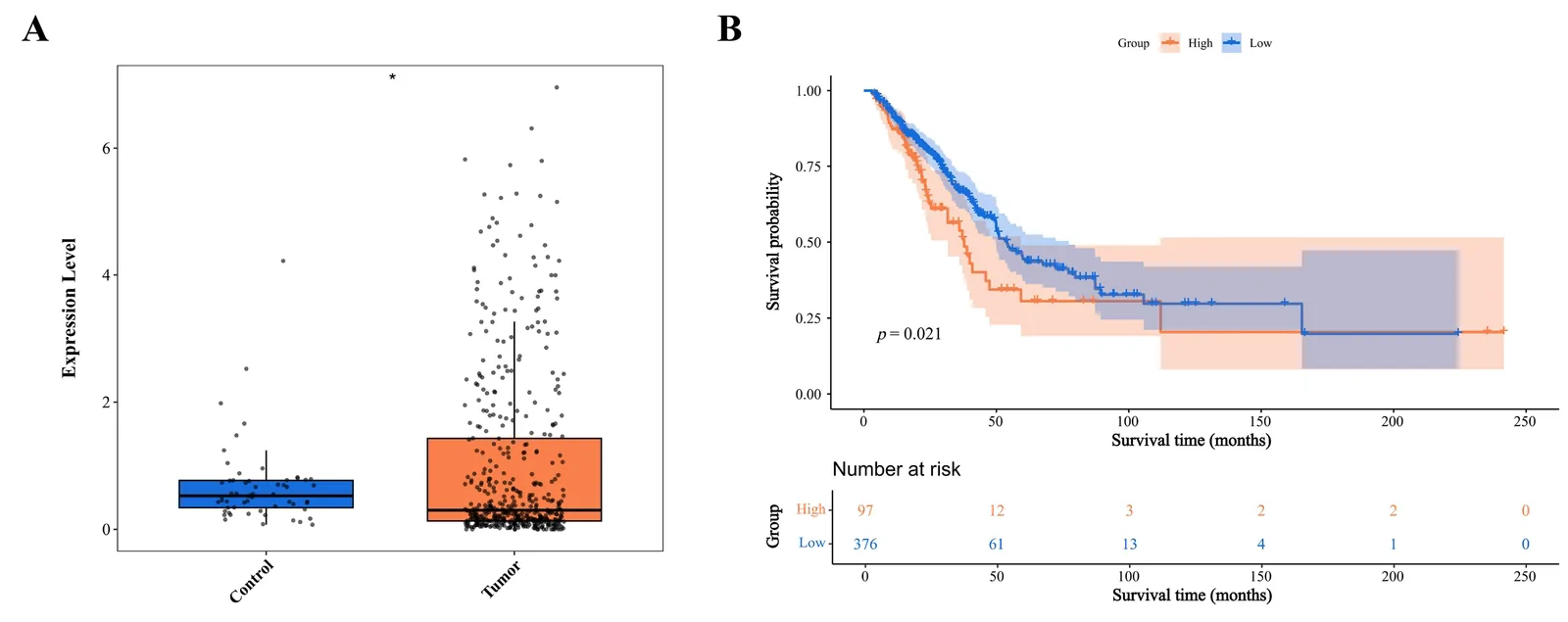
LINC01133 expression differs between LUAD and normal lung tissues and is associated with poor patient survival. (A) LINC01133 expression was compared between LUAD tumor tissues and 58 adjacent normal lung tissues; (B) Relationships between the LINC01133 expression level and the survival rate of LUAD patients. The Wilcoxon rank-sum test was used to compare two groups, and the log-rank test was carried out to analyze survival. ^*^P < 0.05. LUAD: Lung adenocarcinoma.

To determine the clinical significance of LINC01133 in LUAD, the transcriptomic and clinical information of the TCGA-LUAD cohort was obtained from the UCSC Xena database. To minimize the potential influence of non-tumor biological factors, tissues with survival times of less than 90 days were excluded, and 473 LUAD tumor tissue samples were ultimately included for subsequent analysis. LINC01133 expression differed significantly between LUAD and normal lung tissues, with substantial expression heterogeneity and a distinct high-expression subset observed in LUAD tissues [Figure 2A]. Furthermore, Kaplan-Meier survival analysis revealed that LUAD patients with high LINC01133 expression, defined according to the optimal cutoff value of LINC01133 expression, exhibited significantly decreased survival rates compared with those with low LINC01133 expression [Figure 2B]. Consequently, LINC01133 expression is closely associated with unfavorable prognostic outcomes in LUAD patients, suggesting its potential as a biomarker for prognosis prediction and as a therapeutic intervention target.

### LINC01133 regulates cisplatin resistance and malignant behavior in LUAD

**Figure 3:**
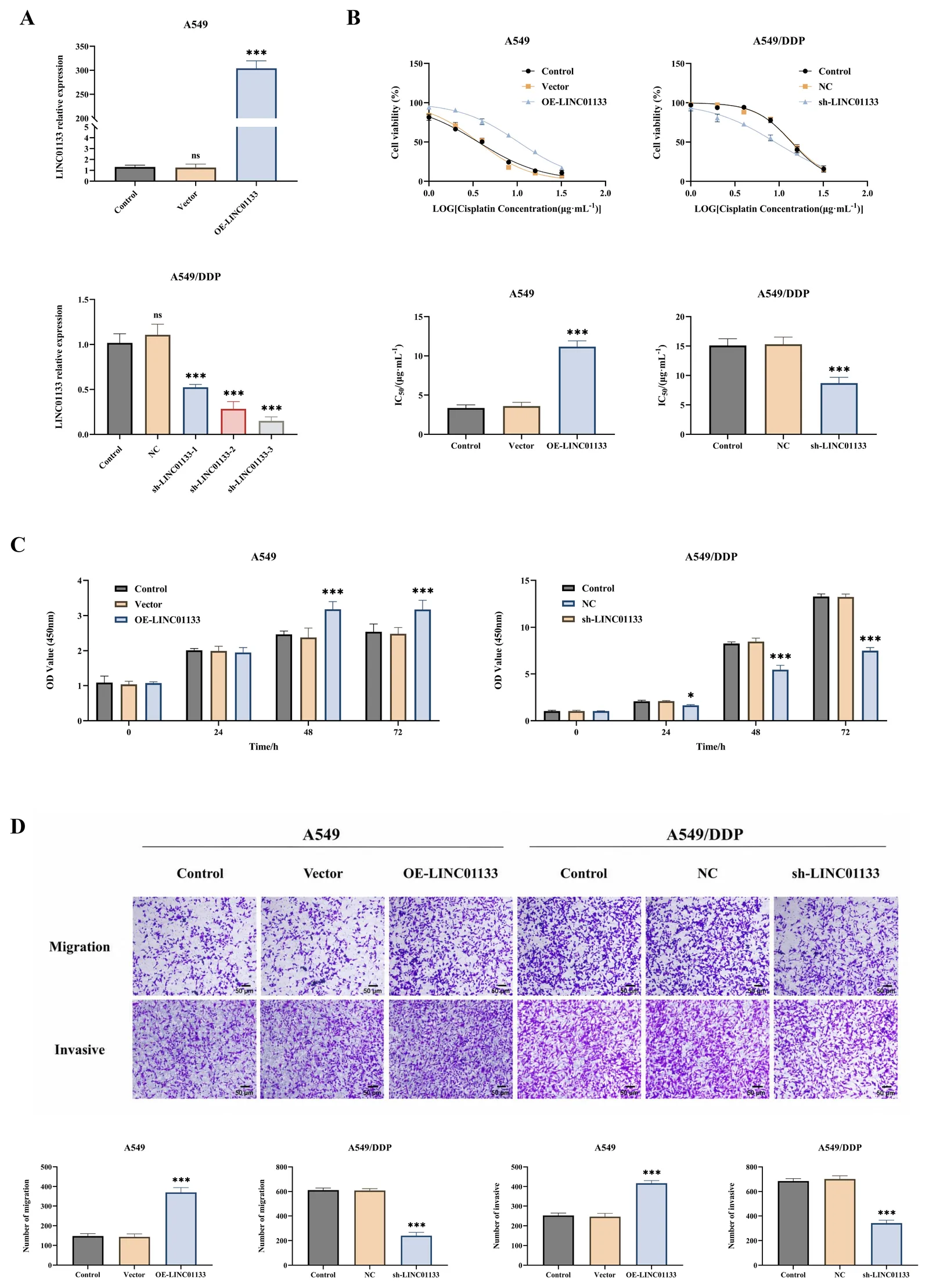
LINC01133 regulates cisplatin resistance and malignant behavior in LUAD. (A) Verification of the relative LINC01133 expression levels in each transfected cell group by qRT-PCR; (B) Effect of LINC01133 expression on cisplatin sensitivity in each cell group, as detected by a CCK-8 assay, with IC_50_ values calculated from dose-response curves; (C) Role of LINC01133 expression in the proliferative capacity of each cell group, as measured through a CCK-8 assay; (D) Role of LINC01133 expression in the migratory and invasive capabilities of every cell group, as measured through a Transwell assay. Scale bar: 50 μm. Nonlinear regression analysis was conducted to fit the dose-response curves. Comparisons among multiple group means were completed with one-way ANOVA. ^*^P < 0.05; ^***^P < 0.001; ns: not significant. LUAD: Lung adenocarcinoma; qRT-PCR: quantitative reverse transcription-polymerase chain reaction; CCK-8: Cell Counting Kit-8; IC_50_: half-maximal inhibitory concentration; ANOVA: analysis of variance; DDP: cisplatin.

To investigate the impact of LINC01133 on LUAD cisplatin resistance, this study successfully constructed stable LINC01133-overexpressing A549 cell lines and stable LINC01133-knockdown A549/DDP cell lines. After confirming altered expression by qRT-PCR, sh-LINC01133-3, which was the most effectively silenced gene, was selected for subsequent experiments [Figure 3A]. According to the results of the CCK-8 assays, LINC01133 overexpression significantly increased cisplatin resistance in A549 cells, whereas LINC01133 knockdown partially reversed the cisplatin-resistant phenotype and significantly increased cisplatin sensitivity in A549/DDP cells [Figure 3B]. Further cellular functional assays confirmed that overexpression of LINC01133 enhanced A549 cell proliferation, invasion, and migration, whereas LINC01133 knockdown effectively suppressed these malignant phenotypes of A549/DDP cells [Figure 3C and D]. Based on the above findings, LINC01133 not only regulates the malignant phenotype of LUAD cells but is also a key factor in maintaining their resistance to cisplatin.

### LINC01133 attenuates cisplatin cytotoxicity by suppressing the Caspase-1/GSDMD pyroptosis pathway

**Figure 4:**
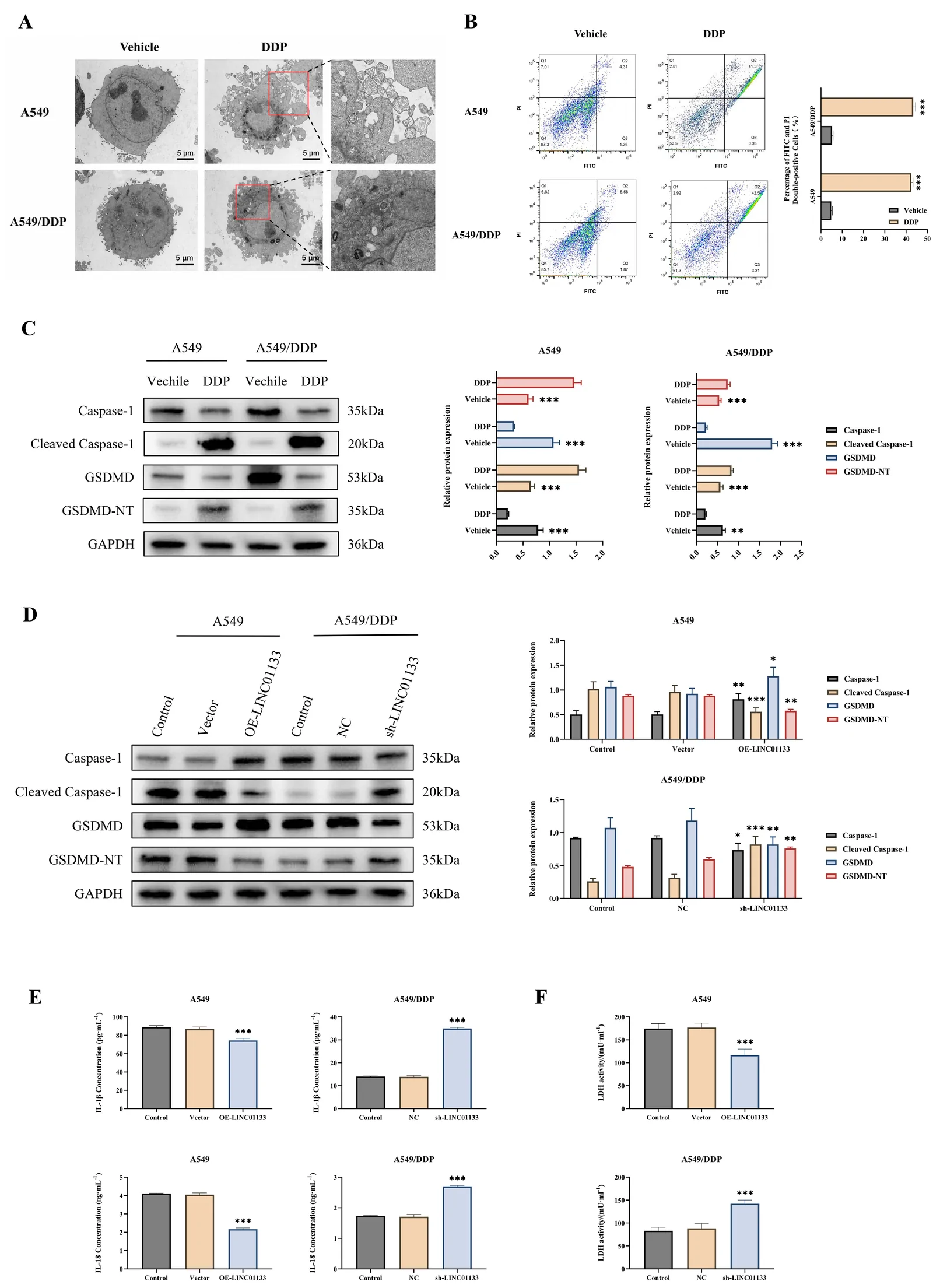
LINC01133 attenuates cisplatin cytotoxicity by suppressing the Caspase-1/GSDMD pyroptosis pathway. (A) Morphological changes in A549 and A549/DDP cells following cisplatin administration were measured by TEM. Scale bar: 5 μm; (B) The proportion of late apoptotic/necrotic cells after cisplatin administration was identified by Annexin V-FITC/PI double staining; (C) Changes in the protein levels of Caspase-1, cleaved Caspase-1, GSDMD, and GSDMD-NT in cells before and after cisplatin treatment were measured by Western blotting. Data were obtained from three independent biological replicates and are presented as the mean ± SD (*n* = 3). Densitometric values of the target proteins were normalized to GAPDH and are presented as relative protein expression levels; (D) Role of LINC01133 expression in key pyroptosis markers, as measured by Western blotting. Data were obtained from three independent biological replicates and are presented as the mean ± SD (*n* = 3). Densitometric values of the target proteins were normalized to GAPDH and are presented as relative protein expression levels; (E) Effect of LINC01133 expression on the levels of the inflammatory cytokines IL-1β and IL-18 in cell supernatants measured via ELISA; (F) Effect of LINC01133 expression on cell membrane leakage, as assessed by LDH release assays. Comparisons among multiple group means were performed by one-way ANOVA. ^*^P < 0.05; ^**^P < 0.01; ^***^P < 0.001. DDP: Cisplatin; TEM: transmission electron microscopy; SD: standard deviation; ELISA: enzyme-linked immunosorbent assay; LDH: lactate dehydrogenase; ANOVA: analysis of variance.

To elucidate the cytotoxic mechanism of cisplatin and the regulatory role of LINC01133 in this process, we systematically analyzed cell death phenotypes and related signaling pathways. TEM revealed that following cisplatin treatment at the IC_50_ for 48 h, both A549 and A549/DDP cells displayed typical features of pyroptosis, including cell swelling, organelle damage, and pyroptotic body formation on the cell membrane surface [Figure 4A]. Annexin V-FITC/PI double staining showed a significant increase in the late apoptotic/necrotic cell proportion after cisplatin treatment, indicating increased late-stage cell death and loss of plasma membrane integrity rather than specifically demonstrating pyroptosis [Figure 4B]. At the molecular level, Western blotting showed that cisplatin effectively activated the Caspase-1/GSDMD axis, as evidenced by the significant upregulation of GSDMD-NT and cleaved Caspase-1 [Figure 4C]. Further experiments confirmed that overexpression of LINC01133 inhibited cisplatin-induced Caspase-1/GSDMD pathway activation in A549 cells, reducing LDH release and secreted IL-1β and IL-18 levels. Conversely, knockdown of LINC01133 restored its activity in A549/DDP cells and upregulated these inflammatory markers [Figure 4D-F]. These findings suggest that the anti-tumor effect of cisplatin occurs partly through the activation of the Caspase-1/GSDMD-mediated pyroptosis pathway, whereas LINC01133 attenuates cisplatin cytotoxicity by inhibiting this pathway, thereby promoting drug resistance.

### LINC01133 regulates cisplatin sensitivity and the Caspase-1/GSDMD pathway across different LUAD cell models

**Figure 5:**
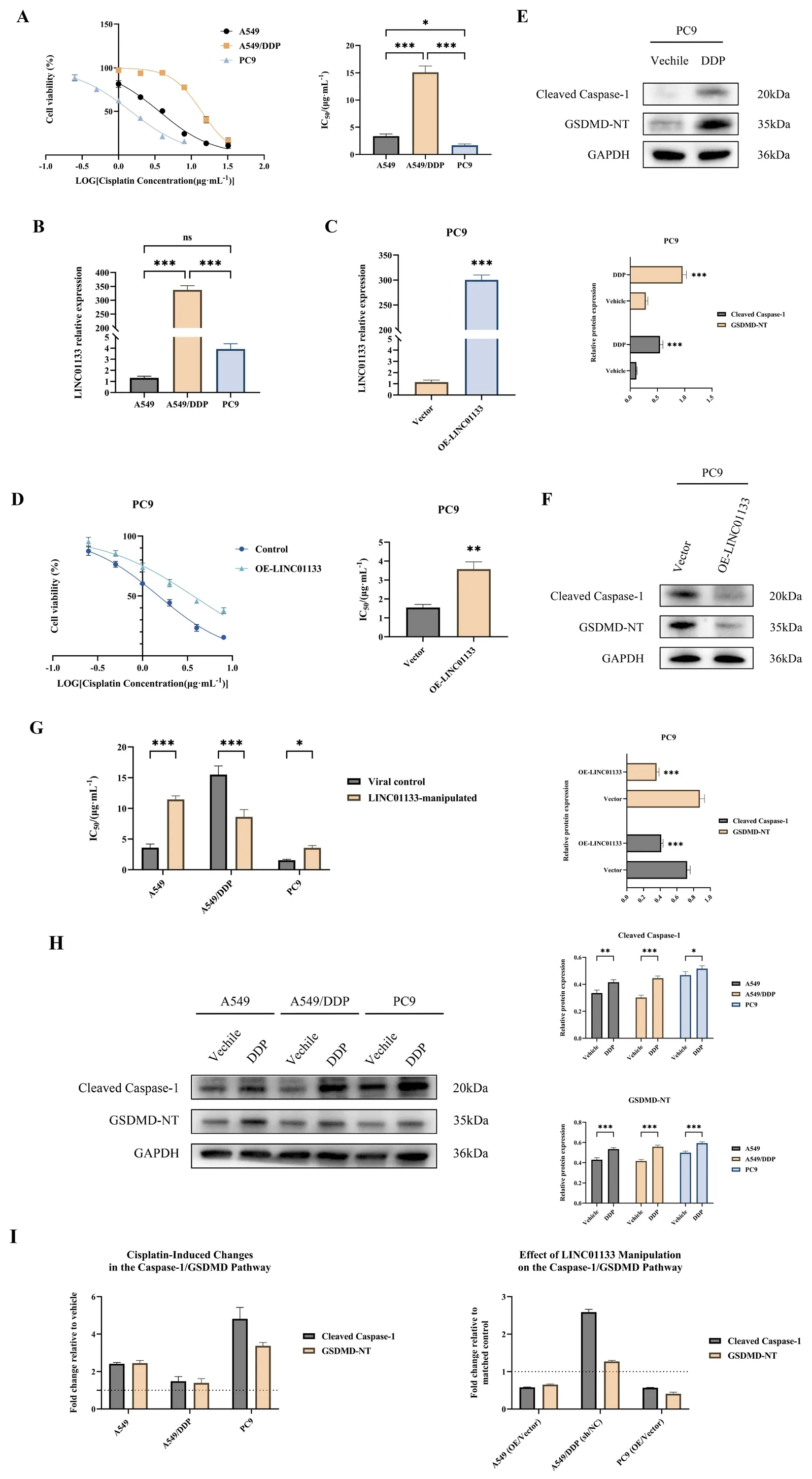
LINC01133 regulates cisplatin sensitivity and the Caspase-1/GSDMD pathway across different LUAD cell models. (A) Cisplatin dose–response curves of A549, A549/DDP, and PC9 cells determined using the CCK-8 assay, together with IC_50_ values calculated by nonlinear regression; (B) Basal LINC01133 expression levels in A549, A549/DDP, and PC9 cells measured by qRT-PCR; (C) The relative expression level of LINC01133 in transfected PC9 cells was determined by qRT-PCR; (D) The effect of LINC01133 expression on the cisplatin sensitivity of PC9 cells was assessed using the CCK-8 assay. Cisplatin concentrations were set at 0.25, 0.5, 1, 2, 4, and 8 μg/mL, and IC_50_ values were calculated from dose-response curves fitted by nonlinear regression; (E) The protein levels of cleaved Caspase-1 and GSDMD-NT in PC9 cells before and after cisplatin treatment were examined by Western blotting. Densitometric values of the target proteins were normalized to GAPDH and are presented as relative protein expression levels. Data were obtained from three independent biological replicates and are presented as the mean ± SD (*n* = 3); (F) The effects of LINC01133 expression on key pyroptosis-related proteins in PC9 cells were evaluated by Western blotting. Densitometric values of the target proteins were normalized to GAPDH and are presented as relative protein expression levels. Data were obtained from three independent biological replicates and are presented as the mean ± SD (*n* = 3); (G) Summary of cisplatin IC_50_ values in the matched viral control and LINC01133-manipulated groups across different LUAD cell models. LINC01133 was overexpressed in A549 and PC9 cells and knocked down in A549/DDP cells. Comparisons were performed within each cellular background; (H) Western blotting and densitometric analysis of cleaved Caspase-1 and GSDMD-NT levels in A549, A549/DDP, and PC9 cells before and after cisplatin treatment; (I) Within-cell-model normalized changes in cleaved Caspase-1 and GSDMD-NT levels. The left panel shows cisplatin-induced changes, whereas the right panel shows the effects of LINC01133 manipulation. LINC01133 was overexpressed in A549 and PC9 cells and knocked down in A549/DDP cells. The dashed line represents a fold change of 1. A fold change greater than 1 indicates an increase in protein level relative to the corresponding matched control, whereas a fold change less than 1 indicates a decrease. Statistical comparisons among three groups were performed using one-way ANOVA followed by Tukey’s multiple-comparisons test, whereas comparisons between two groups were performed using a two-tailed unpaired Student’s *t*-test. ^*^P < 0.05; ^**^P < 0.01; ^***^P < 0.001; ns: not significant. LUAD: Lung adenocarcinoma; DDP: cisplatin; CCK-8: Cell Counting Kit-8; IC_50_: half-maximal inhibitory concentration; qRT-PCR: quantitative reverse transcription-polymerase chain reaction; SD: standard deviation; ANOVA: analysis of variance.

To exclude potential A549 cell-specific effects, PC9 LUAD cells with a distinct genetic background were selected to validate the regulatory effects of LINC01133 on cisplatin sensitivity and the pyroptosis pathway. Cisplatin dose–response analysis showed that PC9 cells exhibited the lowest IC_50_, followed by A549 cells, whereas A549/DDP cells displayed the highest IC_50_. These findings indicate that, under the present experimental conditions, PC9 cells can serve as a relatively cisplatin-sensitive LUAD cell model [Figure 5A]. qRT-PCR analysis showed that the basal expression level of LINC01133 was markedly higher in A549/DDP cells than in A549 and PC9 cells, whereas no significant difference was observed between A549 and PC9 cells [Figure 5B]. PC9-Vector and PC9-OE-LINC01133 cells were subsequently established by lentiviral transduction, and successful LINC01133 overexpression was confirmed by qRT-PCR [Figure 5C]. CCK-8 assays showed that LINC01133 overexpression significantly increased the cisplatin IC_50_ of PC9 cells, indicating reduced cisplatin sensitivity [Figure 5D]. Western blotting demonstrated that cisplatin treatment markedly increased the expression levels of cleaved Caspase-1 and GSDMD-NT in PC9 cells, indicating activation of the Caspase-1/GSDMD pathway [Figure 5E]. In contrast, LINC01133 overexpression reduced the levels of these proteins, suggesting suppression of this pathway [Figure 5F]. A cross-model summary of the independently obtained IC_50_ values showed that LINC01133 overexpression increased the cisplatin IC_50_ in both A549 and PC9 cells, whereas LINC01133 knockdown decreased the IC_50_ in A549/DDP cells [Figure 5G], indicating a consistent direction of regulation of cisplatin sensitivity. Western blotting indicated that cisplatin generally promoted activation of the Caspase-1/GSDMD pathway, although the magnitude of the response differed among the three cell models [Figure 5H]. Analysis of the normalized data showed that cisplatin treatment consistently increased the levels of cleaved Caspase-1 and GSDMD-NT. LINC01133 overexpression reduced the levels of both proteins in A549 and PC9 cells, whereas LINC01133 knockdown exerted the opposite effect in A549/DDP cells [Figure 5I]. Collectively, these findings demonstrate that LINC01133 exerts consistent regulatory effects on cisplatin sensitivity and the Caspase-1/GSDMD pathway across different LUAD cellular backgrounds.

### LINC01133 promotes cisplatin resistance by maintaining mitochondrial structural and functional integrity by sustaining calcium homeostasis

**Figure 6:**
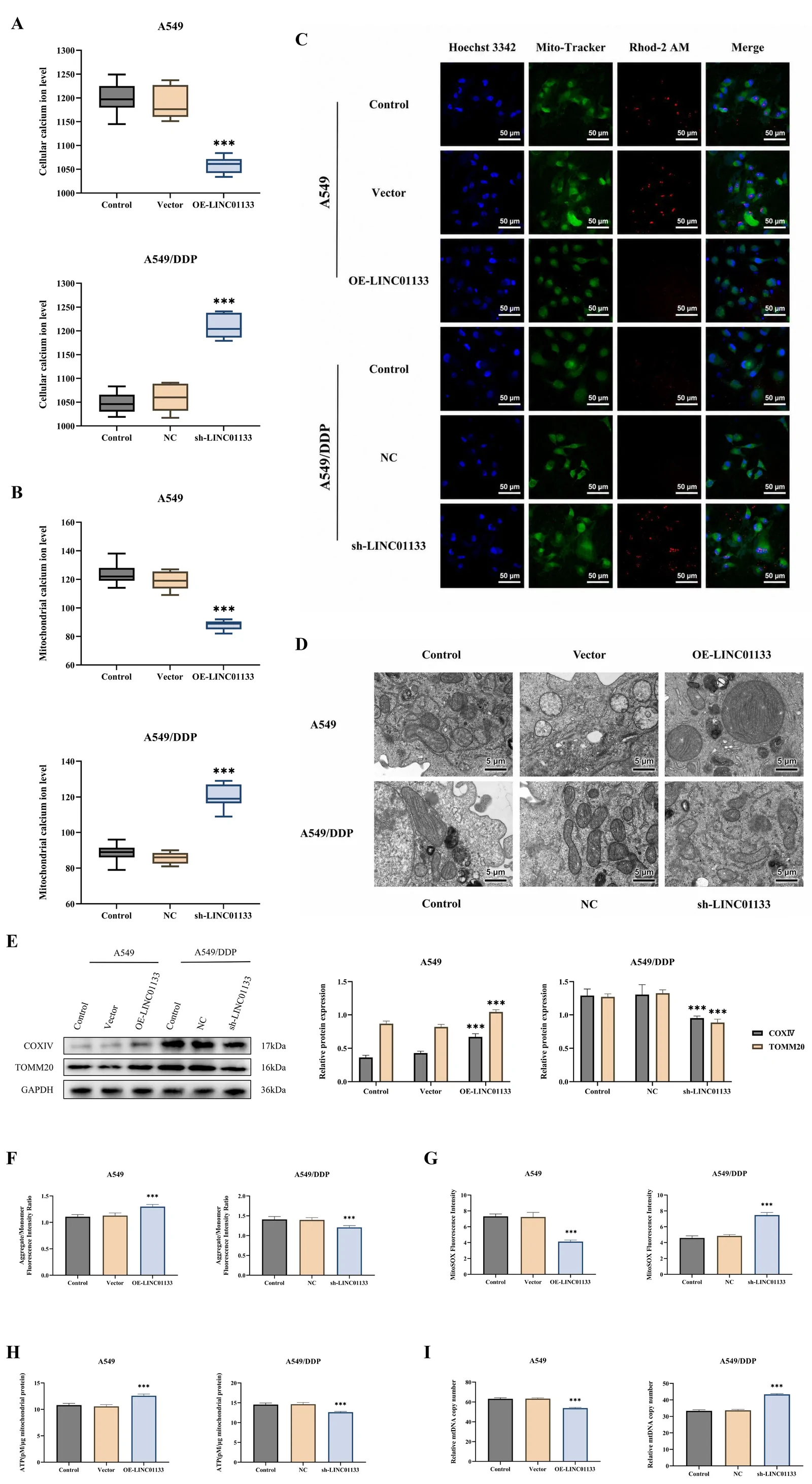
LINC01133 promotes cisplatin resistance by maintaining mitochondrial structural and functional integrity through calcium homeostasis. (A) Effect of LINC01133 expression on the intracellular calcium ion concentration detected using the fluorescent probe Fluo-4 AM; (B) Effect of LINC01133 expression on the mitochondrial calcium ion concentration detected using the Rhod-2 AM fluorescent probe; (C) Role of LINC01133 expression in determining the mitochondrial calcium ion distribution using fluorescence colocalization imaging. Hoechst 33342 was added for nuclear staining (blue), MitoTracker was used for mitochondrial labeling (green), and Rhod-2 AM was used for mitochondrial calcium labeling (red). Scale bar: 50 μm; (D) Role of LINC01133 expression in mitochondrial ultrastructure determined through TEM. Scale bar: 5 μm; (E) Effect of LINC01133 expression on COX IV and TOMM20 protein levels detected by Western blotting. Densitometric values of the target proteins were normalized to GAPDH and are presented as relative protein expression levels. Data were obtained from three independent biological replicates and are presented as the mean ± SD (*n* = 3); (F) Effect of LINC01133 expression on mitochondrial membrane potential assessed using JC-1 staining; (G) Effect of LINC01133 expression on mitochondrial ROS levels detected using MitoSOX staining; (H) Effect of LINC01133 expression on ATP content measured using a chemiluminescence assay; (I) Effect of LINC01133 expression on mtDNA copy number determined by PCR. One-way ANOVA was employed to compare several groups. ^***^P < 0.001. TEM: Transmission electron microscopy; SD: standard deviation; ROS: reactive oxygen species; ATP: adenosine triphospate; mtDNA: mitochondrial DNA; PCR: polymerase chain reaction; ANOVA: analysis of variance; DDP: cisplatin.

To investigate the downstream mechanism through which LINC01133 regulates pyroptosis and drug resistance, we examined changes in mitochondrial structure and function. Staining with Fluo-4 AM and Rhod-2 AM fluorescent probes revealed that LINC01133 knockdown markedly increased the calcium ion concentration in the cytoplasm and mitochondrial matrix, indicating “calcium overload” [Figure 6A and B]. Fluorescence colocalization imaging revealed that in cells with evident pyroptosis, the mitochondrial calcium signal was enhanced and highly colocalized with the mitochondrial marker [Figure 6C]. TEM revealed that calcium overload was accompanied by severe damage to mitochondrial ultrastructure, including mitochondrial swelling, blurred boundaries, disrupted cristae, and vacuolization [Figure 6D]. Western blotting revealed that both inner and outer mitochondrial membrane markers (COX IV and TOMM20, respectively) were downregulated in the calcium overload group but upregulated in the LINC01133 overexpression group [Figure 6E]. Additionally, functional assays confirmed that interference with LINC01133 expression lowered mitochondrial membrane potential, increased mitochondrial ROS levels, reduced ATP content, and increased mtDNA copy number, whereas LINC01133 overexpression reversed these dysfunctions [Figure 6F-I]. These results suggest that LINC01133, by inhibiting the pyroptosis pathway, alleviates GSDMD-NT-mediated calcium influx^[22]^ and maintains mitochondrial calcium homeostasis, thereby protecting mitochondrial structural integrity and normal function and ultimately promoting cisplatin resistance in LUAD.

### LINC01133 regulates cisplatin resistance, pyroptosis, and calcium homeostasis through GSDMD

**Figure 7:**
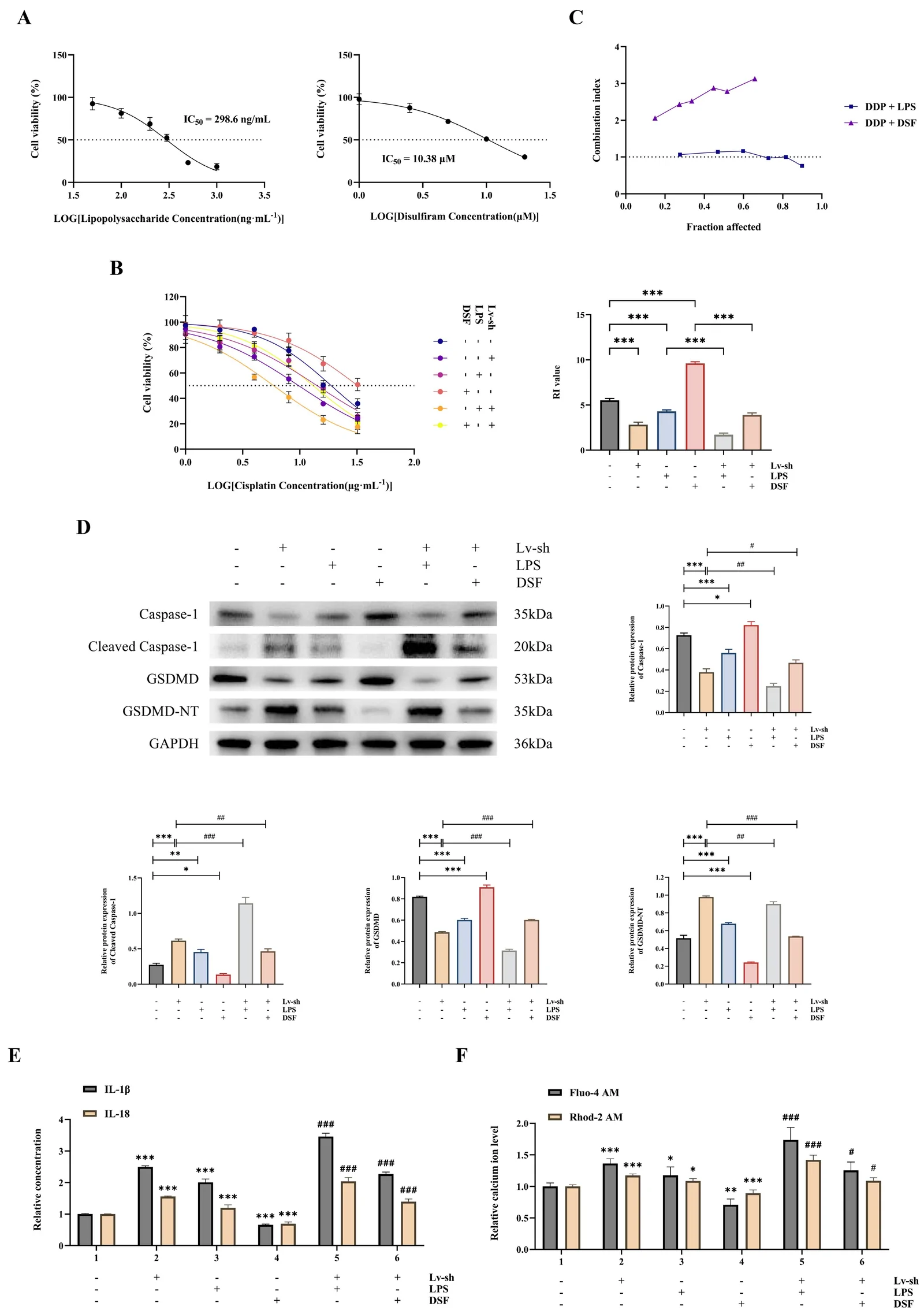
LINC01133 regulates cisplatin resistance, pyroptosis, and calcium homeostasis through GSDMD. (A) A549/DDP cell viability after treatment with varying doses of LPS (50, 100, 200, 300, 500, and 1,000 ng/mL) and DSF (1, 2.5, 5, 10, and 20 μM) was measured through a CCK-8 assay to determine the optimal working concentrations; (B) Changes in the cisplatin RI of A549/DDP cells after silencing LINC01133 and modulating GSDMD expression, as measured by a CCK-8 assay; (C) The combination effects of DDP with LPS or DSF were quantitatively analyzed using the Chou-Talalay model, and CI-Fa curves were generated. Fa represents the effect level; CI < 1 indicates synergism, CI = 1 indicates an additive effect, and CI > 1 indicates antagonism; (D) Changes in the protein expression of key pyroptosis markers detected by Western blotting. Densitometric values of the target proteins were normalized to GAPDH and are presented as relative protein expression levels. Data were obtained from three independent biological replicates and are presented as the mean ± SD (*n* = 3); (E) The levels of the inflammatory factors IL-1β and IL-18 in the cell supernatants were analyzed by ELISA; (F) Changes in cytoplasmic and mitochondrial calcium ion levels were detected using Fluo-4 AM and Rhod-2 AM fluorescent probes, respectively. Comparisons among multiple group means were conducted using two-way ANOVA. ^*^P < 0.05, ^**^P < 0.01, and ^***^P < 0.001 indicate statistical significance *vs.* the A549/DDP-NC group; ^#^*P* < 0.05, ^##^*P* < 0.01, and ^###^*P* < 0.001 indicate statistical significance *vs.* the A549/DDP-sh-LINC01133 group. DDP: Cisplatin; LPS: lipopolysaccharide; DSF: disulfiram; CCK-8: Cell Counting Kit-8; RI: resistance index; SD: standard deviation; ELISA: enzyme-linked immunosorbent assay; ANOVA: analysis of variance; IC_50_: half-maximal inhibitory concentration.

To elucidate the key effect of GSDMD on LUAD cell resistance to cisplatin, rescue experiments were conducted in A549/DDP cells. First, concentrations were determined by assessing cell viability after treatment with the GSDMD pathway activator LPS and inhibitor DSF, which were found to be 300 ng/mL and 10 μM, respectively [Figure 7A]. CCK-8 assays revealed that both silencing LINC01133 and LPS treatment improved cisplatin sensitivity in A549/DDP cells, as indicated by a decreased RI. Conversely, DSF intervention had the opposite effect, partially restoring the resistant phenotype. The results of the combination treatment experiments revealed that DSF partially attenuated the cisplatin-sensitizing effect induced by LINC01133 silencing, while LPS further strengthened this effect [Figure 7B]. The combined effects of DDP with LPS or DSF were quantitatively analyzed using the Chou-Talalay model. The results showed that the combination of DDP and LPS produced an additive effect, with a tendency toward synergism at higher effect levels, whereas the combination of DDP and DSF exhibited a stable antagonistic effect [Figure 7C]. These findings further support, at a quantitative level, the conclusion that activation of the GSDMD pathway enhances cisplatin cytotoxicity, whereas its inhibition reduces cisplatin sensitivity. At the molecular level, the Western blot results were consistent with the observed phenotypic changes: silencing LINC01133 and LPS treatment promoted Caspase-1 activation and enhanced GSDMD cleavage, as evidenced by increased cleaved Caspase-1 and GSDMD-NT levels, whereas DSF intervention inhibited the activation and cleavage of these pyroptosis-related proteins [Figure 7D]. ELISA results further supported the above regulatory pattern [Figure 7E]. Further analysis revealed that silencing LINC01133 and activating GSDMD increased the levels of calcium ions in the cytoplasm and mitochondria (calcium overload), whereas DSF treatment significantly reversed these effects [Figure 7F]. In summary, LINC01133 may regulate calcium homeostasis by modulating GSDMD-mediated pyroptosis, thereby contributing to the maintenance of cisplatin resistance in LUAD cells.

### *In vivo* validation that LINC01133 promotes cisplatin resistance through suppressing pyroptosis

**Figure 8:**
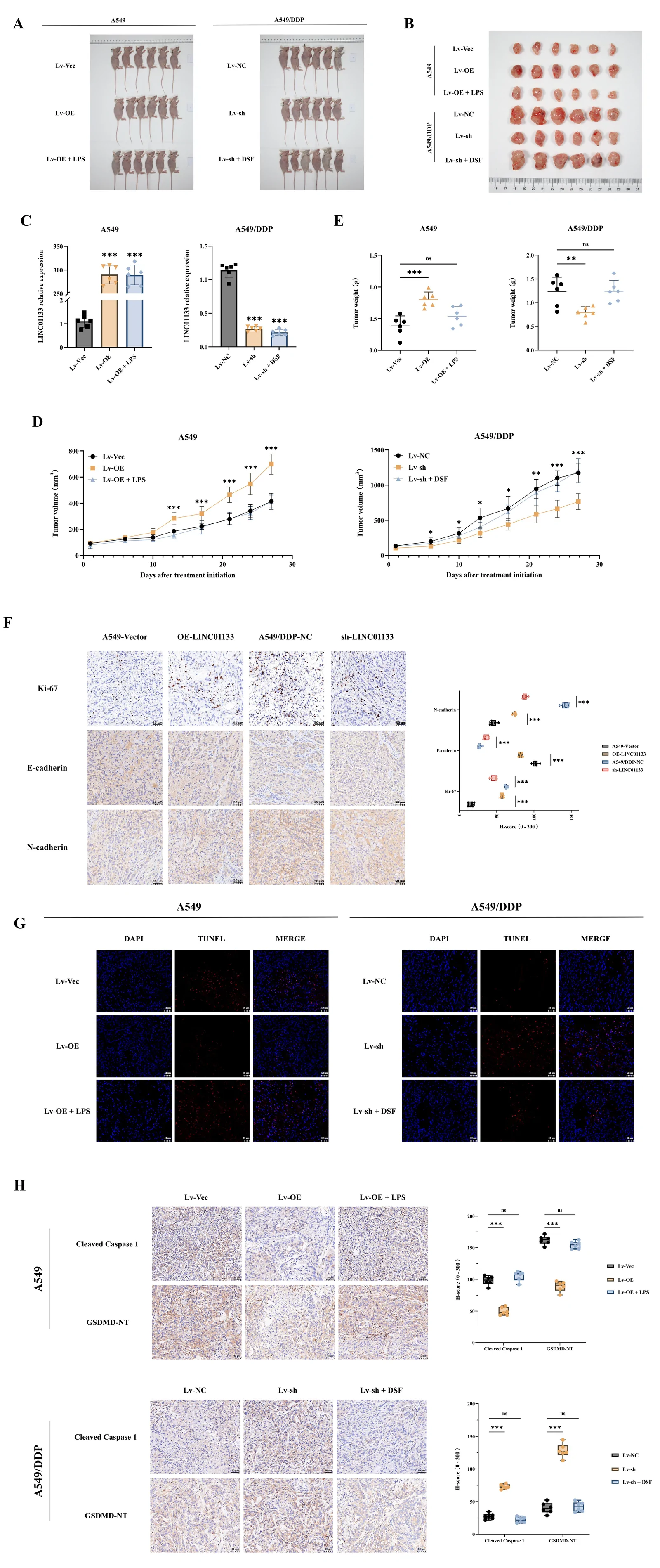
*In vivo* validation that LINC01133 promotes cisplatin resistance by inhibiting pyroptosis. (A) Tumor sizes in each group of BALB/c nude mice; (B) Excised tumor tissues from each group immediately after the experiment; (C) LINC01133 levels in tumor tissues measured by qRT-PCR; (D) Tumor growth curves plotted based on measured tumor volumes; (E) Comparison of final tumor weights of BALB/c nude mice across diverse groups; (F) IHC detection of Ki-67, E-cadherin, and N-cadherin expression in tumor tissues from diverse groups (20×). Scale bar: 50 μm; (G) TUNEL-positive cell death in tumor tissues detected by TUNEL staining (20×). Scale bar: 50 μm; (H) IHC detection of cleaved Caspase-1 and GSDMD-NT levels in tumor tissues from diverse groups (20×). Scale bar: 50 μm. Comparisons among multiple group means were performed with one-way ANOVA. ^*^P < 0.05; ^**^P < 0.01; ^***^P < 0.001; ns: not significant. qRT-PCR: Quantitative reverse transcription-polymerase chain reaction; IHC: immunohistochemical; TUNEL: transferase dUTP nick-end labeling; ANOVA: analysis of variance; DDP: cisplatin; LPS: lipopolysaccharide; DSF: disulfiram.

To validate the above mechanism *in vivo*, a BALB/c nude mouse xenograft tumor model was constructed in six experimental groups: Lv-A549-Vector, Lv-A549-OE-LINC01133, Lv-A549-OE-LINC01133 + LPS, Lv-A549/DDP-NC, Lv-A549/DDP-sh-LINC01133, and Lv-A549/DDP-sh-LINC01133 + DSF. The tumor tissues were excised immediately after the experiment [Figure 8A and B]. qRT-PCR results showed that LINC01133 expression in tumor tissues in each group was as expected, confirming successful construction of the animal model [Figure 8C]. Compared with those in the Lv-Vector group, tumor growth was significantly accelerated, and the final tumor weight was increased in the Lv-OE group; the combined use of LPS restored the tumor-suppressive effect of cisplatin. Conversely, relative to those in the Lv-NC group, tumor growth was inhibited, and the final tumor weight was reduced in the Lv-sh group; moreover, DSF administration attenuated the tumor-suppressive effect of cisplatin [Figure 8D and E]. The immunohistochemistry results indicated that, compared with the Lv-vector group, the Lv-OE group exhibited increased expression of Ki-67 and N-cadherin, whereas E-cadherin expression was decreased. In contrast, compared with the Lv-NC group, the Lv-sh group showed the opposite expression pattern for these markers [Figure 8F]. LINC01133 overexpression reduced TUNEL positivity and the expression of these pyroptosis-related proteins, and these effects were reversed by LPS; conversely, LINC01133 knockdown produced the opposite changes, which were attenuated by DSF [Figure 8G and H]. In summary, the results of the present study confirmed in an animal model that LINC01133 promotes malignant phenotypes including LUAD cell proliferation, invasion, and migration. The inhibition of the Caspase-1/GSDMD pathway-induced pyroptosis weakens the tumor-suppressive effect of cisplatin, thereby promoting cisplatin resistance in LUAD.

## DISCUSSION

LINC01133, a lncRNA with dual regulatory characteristics, has been confirmed to regulate malignant behaviors including cell proliferation, invasion, and migration in various tumors^[23-25]^. In NSCLC, LINC01133 can bind to epigenetic regulators such as EZH2 and LSD1, inhibiting the transcription of tumor suppressor genes (such as KLF2 and P21), thereby playing an oncogenic role^[26]^. Building on these findings, the results of the present study reveal a new activity *in vitro* and *in vivo*: in A549/DDP cells, LINC01133 is specifically enriched in mitochondria and confers cisplatin tolerance to tumor cells through the inhibition of Caspase-1/GSDMD-mediated pyroptosis, thereby blocking GSDMD-NT pore-induced mitochondrial calcium overload and dysfunction. Silencing LINC01133 restored the cytotoxicity of cisplatin to LUAD cells and inhibited their malignant behaviors. These findings not only confirm the oncogenic properties of LINC01133 but also expand its functional understanding from merely regulating malignant behaviors to being involved in chemotherapy resistance, suggesting that it is a candidate anti-LUAD therapeutic target.

Cell death includes diverse forms, such as apoptosis, pyroptosis, necrosis, ferroptosis, and autophagy. Previous studies have often attributed the anti-tumor effects of chemotherapeutic drugs to their ability to induce apoptosis in tumor cells. However, accumulating evidence has indicated that pyroptosis, which is a form of programmed cell death mediated by the gasdermin protein family, has an important effect on the antitumor effects of chemotherapy^[27-29]^. The Caspase-1/GSDMD pathway accounts for the classical route through which pyroptosis is mediated: after the inflammasome is activated, Caspase-1 can be recruited and activated, which subsequently cleaves GSDMD, leading to the release of its N-terminal fragment (GSDMD-NT) to the inner leaflet of the plasma membrane. GSDMD-NT creates pores on the cell membrane, ultimately causing cell rupture and the release of inflammatory factors^[30,31]^. This study first confirmed that cisplatin can effectively activate the classical Caspase-1/GSDMD pyroptosis pathway in LUAD cells. Moreover, in cells with high LINC01133 expression, this pyroptosis pathway was significantly inhibited, as indicated by the downregulation of cleaved Caspase-1 and GSDMD-NT levels, accompanied by reduced LDH release and decreased IL-1β and IL-18 production. Functional rescue experiments further confirmed that the GSDMD inhibitor DSF could mimic the LINC01133 overexpression phenotype, whereas the GSDMD agonist LPS could partially reverse the sensitizing effect induced by LINC01133 knockdown, directly demonstrating that GSDMD-mediated pyroptosis is a key downstream effector of LINC01133.

To further determine whether the regulatory effects of LINC01133 on cisplatin sensitivity and the Caspase-1/GSDMD pathway are robust beyond the A549 cellular context, PC9 cells with a distinct genetic background were included as an additional validation model. PC9 is a widely used lung cancer cell line with molecular characteristics that differ substantially from those of A549 cells and is commonly employed as a cisplatin-sensitive parental model in studies of acquired drug resistance. Under the present experimental conditions, PC9 cells could be classified as a relatively cisplatin-sensitive model. LINC01133 overexpression reduced the sensitivity of PC9 cells to cisplatin and suppressed cisplatin-induced activation of the Caspase-1/GSDMD pathway, consistent with the regulatory pattern observed in A549 cells, which also represent a cisplatin-sensitive parental model. Because each cell model was treated with its own IC_50_ concentration of cisplatin and the corresponding experiments were conducted independently in different batches, protein expression changes were normalized to the matched control within each cellular background to assess the direction of LINC01133-mediated regulation across different cell models. The regulatory effects of LINC01133 were directionally consistent across all three cellular models. In A549 and PC9 cells, LINC01133 upregulation reduced cisplatin responsiveness and suppressed activation of the Caspase-1/GSDMD pathway, whereas LINC01133 knockdown in A549/DDP cells produced the opposite effects. Notably, the basal expression level of LINC01133 was significantly higher in A549/DDP cells than in A549 and PC9 cells, whereas no significant difference was observed between the two parental cell lines. This expression pattern suggests that LINC01133 upregulation is more closely associated with the acquired cisplatin-resistant phenotype than with intrinsic molecular heterogeneity among different LUAD cell lines. Nevertheless, differences in basal cisplatin sensitivity among PC9, A549, and A549/DDP cells also indicate that the genetic background of each cell line and other resistance-associated mechanisms may act in concert with LINC01133 to determine overall drug response. Collectively, these findings indicate that the regulatory effects of LINC01133 on cisplatin responsiveness and the Caspase-1/GSDMD pathway are not specific to A549 cells but can be reproduced in another LUAD model with a distinct genetic background, further supporting the robustness and cross-cellular applicability of this regulatory mechanism.

The formation of GSDMD-NT pores can disrupt the intracellular ion balance and remodel transmembrane ion homeostasis^[31-33]^. The dynamic balance of calcium ions, which are crucial second messengers, is closely related to cell fate. Imbalance in mitochondria, which are the core regulators of intracellular calcium homeostasis, has been proven to be a critical link in the induction of pyroptosis and various pathological states^[34-36]^. In cells with low LINC01133 expression (i.e., in a pyroptosis-activated state), calcium ion concentrations in both the cytoplasm and mitochondrial matrix were significantly elevated, indicating a typical phenomenon of mitochondrial calcium overload. This likely originates from extracellular calcium influx mediated by GSDMD-NT pores and excessive uptake by mitochondria^[22,37]^. Systematic evaluation of mitochondrial structure and function consistently revealed that calcium overload caused severe damage: TEM revealed mitochondrial swelling and disrupted cristae, and Western blot analysis revealed decreased expression of inner and outer mitochondrial membrane marker proteins, suggesting impaired structural integrity; at the functional level, further observations indicated reduced membrane potential, elevated ROS content, decreased ATP synthesis, and increased mtDNA copy number. Taken together, these findings reveal that LINC01133, by inhibiting the pyroptosis pathway, blocks the formation of GSDMD-NT-mediated membrane pores, thereby alleviating mitochondrial calcium overload, maintaining mitochondrial functional integrity, and promoting tumor cell survival upon chemotherapeutic stress.

Based on these findings, we speculate that there might be a vicious self-amplifying cycle between pyroptosis pathway activation and mitochondrial functional impairment. On the one hand, GSDMD-NT pore-triggered mitochondrial calcium overload can directly lead to structural and functional collapse. On the other hand, impaired mitochondria can secrete damage-associated molecular patterns (DAMPs), including ROS and mtDNA, which in turn activate inflammasomes, further exacerbating the activation of pyroptosis signaling pathways^[38-40]^. This positive feedback loop of “pyroptosis-mitochondrial damage-inflammasome reactivation” may have an important effect on the process through which tumor cells continuously adapt to chemotherapeutic stress and ultimately establish a stable drug-resistant phenotype. Notably, our experiments showed that basal GSDMD expression was significantly higher in A549/DDP cells than in A549 cells, suggesting that drug-resistant cells may be in a “pyroptosis-ready state”, whereas high LINC01133 expression inhibits the final execution of pyroptosis. This phenomenon warrants further in-depth investigation: Does LINC01133 participate in resistance formation by regulating the transcription, translation, or post-translational modification of GSDMD? Is GSDMD itself a direct or indirect target of LINC01133? These questions need to be addressed in subsequent studies.

Our findings provide multiple translational approaches for the clinical intervention of cisplatin resistance in LUAD: the mitochondria-enriched LINC01133 can be used as a predictive biomarker for cisplatin sensitivity, with its expression levels detected to stratify patients and formulate personalized chemotherapy regimens; it can also serve as a core therapeutic target, where antisense oligonucleotides and small interfering RNAs targeting and silencing LINC01133 are developed and combined with mitochondria-targeted delivery systems to restore the Caspase-1/GSDMD pyroptosis pathway for reversing drug resistance; meanwhile, combination therapeutic strategies can be designed based on the LINC01133-Caspase-1/GSDMD-Ca^2+^ axis, such as combining cisplatin with GSDMD pathway agonists or calcium homeostasis modulators, warrant further investigation to enhance the anti-tumor effect of cisplatin. Notably, the nude mouse xenograft model used in this study lacks intact T-cell-mediated immunity and therefore cannot directly evaluate pyroptosis-induced anti-tumor immune responses. Thus, whether LINC01133-mediated pyroptosis regulation contributes to anti-tumor immune activation requires further validation in immunocompetent models and may represent a potential direction for future combination immunotherapy studies. In the future, we intend to further validate the function of LINC01133 in multiple cell lines, patient-derived xenograft (PDX) models and syngeneic mouse models, and collect large-sample clinical tissue samples and follow-up data to verify its value as a biomarker.

Although this study has obtained novel findings, several limitations exist. First, although the key findings were further validated in PC9 cells, cross-cell-line validation was limited to an overexpression model and selected endpoints. Further validation using additional LUAD cell lines, knockdown or rescue strategies, and PDX models is warranted. Second, the direct molecular mechanism of LINC01133 in regulating the Caspase-1/GSDMD pathway remains unclear, and its mitochondrial binding partners and regulatory mode need further exploration. Third, the nude mouse xenograft model lacks a complete immune system and cannot reflect the interaction in the tumor microenvironment; syngeneic mouse models should be used in the future. Fourth, the clinical analysis is limited to the TCGA-LUAD cohort without large-scale sample validation, and the correlation of LINC01133 expression with cisplatin efficacy and clinicopathological parameters has not been explored.

In conclusion, this study provides evidence that the mitochondria-associated lncRNA LINC01133 drives the development of cisplatin resistance in LUAD by inhibiting Caspase-1/GSDMD axis-mediated pyroptosis and maintaining mitochondrial functional integrity. This discovery reveals a new mechanism of chemotherapy resistance, lays an important theoretical foundation and provides candidate targets for developing therapeutic strategies to reverse resistance by targeting LINC01133 and its downstream pathways.

## DECLARATIONS

### Availability of data and materials

All data generated or analyzed during this study are available from the corresponding author for reasonable requests.

### Copyright

© The Author(s) 2026.

## References

[1] Han B, Zheng R, Zeng H (2024). Cancer incidence and mortality in China, 2022. J Natl Cancer Cent.

[2] Sung H, Ferlay J, Siegel RL (2021). Global cancer statistics 2020: GLOBOCAN estimates of incidence and mortality worldwide for 36 cancers in 185 countries. CA Cancer J Clin.

[3] Fennell DA, Summers Y, Cadranel J (2016). Cisplatin in the modern era: the backbone of first-line chemotherapy for non-small cell lung cancer. Cancer Treat Rev.

[4] (2015). Garon EB, Rizvi NA, Hui R, et al.; KEYNOTE-001 Investigators. Pembrolizumab for the treatment of non-small-cell lung cancer. N Engl J Med.

[5] Hellmann MD, Paz-Ares L, Bernabe Caro R (2019). Nivolumab plus ipilimumab in advanced non-small-cell lung cancer. N Engl J Med.

[6] Szejniuk WM, Cekala M, Bøgsted M (2021). Adjuvant platinum-based chemotherapy in non-small cell lung cancer: the role of relative dose-intensity and treatment delay. Cancer Treat Res Commun.

[7] Lv P, Man S, Xie L, Ma L, Gao W (2021). Pathogenesis and therapeutic strategy in platinum resistance lung cancer. Biochim Biophys Acta Rev Cancer.

[8] Min HY, Lee HY (2021). Mechanisms of resistance to chemotherapy in non-small cell lung cancer. Arch Pharm Res.

[9] Du H, Xu T, Yu S, Wu S, Zhang J (2025). Mitochondrial metabolism and cancer therapeutic innovation. Signal Transduct Target Ther.

[10] Jin P, Jiang J, Zhou L (2022). Mitochondrial adaptation in cancer drug resistance: prevalence, mechanisms, and management. J Hematol Oncol.

[11] Zong Y, Li H, Liao P (2024). Mitochondrial dysfunction: mechanisms and advances in therapy. Signal Transduct Target Ther.

[12] Galluzzi L, Senovilla L, Vitale I (2012). Molecular mechanisms of cisplatin resistance. Oncogene.

[13] Cocetta V, Ragazzi E, Montopoli M (2019). Mitochondrial involvement in cisplatin resistance. Int J Mol Sci.

[14] Deng M, Zhou Z, Chen J (2025). Enhanced oxidative phosphorylation driven by TACO1 mitochondrial translocation promotes stemness and cisplatin resistance in bladder cancer. Adv Sci.

[15] Hao Y, Zhou Z, Liu R (2025). Mitochondria-localized MBD2c facilitates mtDNA transcription and drug resistance. Nat Chem Biol.

[16] Kleih M, Böpple K, Dong M (2019). Direct impact of cisplatin on mitochondria induces ROS production that dictates cell fate of ovarian cancer cells. Cell Death Dis.

[17] Lv GY, Mu WT, Cao YN (2025). Cisplatin-induced disruption of mitochondrial divisome leads to enhanced cisplatin resistance in cholangiocarcinoma. J Hepatol.

[18] (2023). Gallo Cantafio ME, Torcasio R, Viglietto G, Amodio N. Non-coding RNA-dependent regulation of mitochondrial dynamics in cancer pathophysiology. Noncoding RNA.

[19] Piergentili R, Sechi S (2024). Non-coding RNAs of mitochondrial origin: roles in cell division and implications in cancer. Int J Mol Sci.

[20] Zhang Y (2024). LncRNA-encoded peptides in cancer. J Hematol Oncol.

[21] Liu MZ, Shao XY, Wu SH (2025). Mitophagy suppression via lncRNA H19 silencing: a novel strategy to overcome cisplatin resistance in lung adenocarcinoma. Cell Cycle.

[22] (2019). de Vasconcelos NM, Van Opdenbosch N, Van Gorp H, Parthoens E, Lamkanfi M. Single-cell analysis of pyroptosis dynamics reveals conserved GSDMD-mediated subcellular events that precede plasma membrane rupture. Cell Death Differ.

[23] Ghafouri-Fard S, Khoshbakht T, Hussen BM, Taheri M, Mokhtari M (2022). A review on the role of LINC01133 in cancers. Cancer Cell Int.

[24] Jiang S, Zhang Q, Li J (2022). New sights into long non-coding RNA LINC01133 in cancer. Front Oncol.

[25] Li Z, Xu D, Chen X, Li S, Chan MTV, Wu WKK (2020). LINC01133: an emerging tumor-associated long non-coding RNA in tumor and osteosarcoma. Environ Sci Pollut Res Int.

[26] Zang C, Nie FQ, Wang Q (2016). Long non-coding RNA LINC01133 represses KLF2, P21 and E-cadherin transcription through binding with EZH2, LSD1 in non small cell lung cancer. Oncotarget.

[27] Su P, Mao X, Ma J (2023). ERRα promotes glycolytic metabolism and targets the NLRP3/caspase-1/GSDMD pathway to regulate pyroptosis in endometrial cancer. J Exp Clin Cancer Res.

[28] Xiang C, Chen L, Zhu S (2024). CRLF1 bridges AKT and mTORC2 through SIN1 to inhibit pyroptosis and enhance chemo-resistance in ovarian cancer. Cell Death Dis.

[29] Zhao K, Wang X, Jin Y (2024). LncRNA ZNF674-AS1 drives cell growth and inhibits cisplatin-induced pyroptosis via up-regulating CA9 in neuroblastoma. Cell Death Dis.

[30] Shi J, Zhao Y, Wang K (2015). Cleavage of GSDMD by inflammatory caspases determines pyroptotic cell death. Nature.

[31] Sborgi L, Rühl S, Mulvihill E (2016). GSDMD membrane pore formation constitutes the mechanism of pyroptotic cell death. EMBO J.

[32] Liu X, Zhang Z, Ruan J (2016). Inflammasome-activated gasdermin D causes pyroptosis by forming membrane pores. Nature.

[33] Kambara H, Liu F, Zhang X (2018). Gasdermin D exerts anti-inflammatory effects by promoting neutrophil death. Cell Rep.

[34] Rizzuto R, Pinton P, Carrington W (1998). Close contacts with the endoplasmic reticulum as determinants of mitochondrial Ca^2+^ responses. Science.

[35] Berridge MJ, Lipp P, Bootman MD (2000). The versatility and universality of calcium signalling. Nat Rev Mol Cell Biol.

[36] Dey K, Bazala MA, Kuznicki J (2020). Targeting mitochondrial calcium pathways as a potential treatment against Parkinson’s disease. Cell Calcium.

[37] Broz P, Pelegrín P, Shao F (2020). The gasdermins, a protein family executing cell death and inflammation. Nat Rev Immunol.

[38] Shimada K, Crother TR, Karlin J (2012). Oxidized mitochondrial DNA activates the NLRP3 inflammasome during apoptosis. Immunity.

[39] Wang Y, Shi P, Chen Q (2019). Mitochondrial ROS promote macrophage pyroptosis by inducing GSDMD oxidation. J Mol Cell Biol.

[40] Ning C, Gao F, Wang Z (2025). LBH589 reduces oxidized mitochondrial DNA and suppresses NLRP3 inflammasome activation to relieve pulmonary inflammation. PLoS One.

